# Parsing Social Network Survey Data from Hidden Populations Using Stochastic Context-Free Grammars

**DOI:** 10.1371/journal.pone.0006777

**Published:** 2009-09-07

**Authors:** Art F. Y. Poon, Kimberly C. Brouwer, Steffanie A. Strathdee, Michelle Firestone-Cruz, Remedios M. Lozada, Sergei L. Kosakovsky Pond, Douglas D. Heckathorn, Simon D. W. Frost

**Affiliations:** 1 Division of Comparative Pathology and Medicine, University of California San Diego, La Jolla, California, United States of America; 2 Division of Global Public Health, Department of Medicine, University of California San Diego, La Jolla, California, United States of America; 3 Patronato proCOMUSIDA, Tijuana, Baja California, México; 4 Division of Infectious Diseases and Division of Biomedical Informatics, School of Medicine, University of California San Diego, La Jolla, California, United States of America; 5 Department of Sociology, Cornell University, Ithaca, New York, United States of America; 6 Laboratory of Viral Zoonotics, Department of Veterinary Medicine, University of Cambridge, Cambridge, United Kingdom; Yale University, United States of America

## Abstract

**Background:**

Human populations are structured by social networks, in which individuals tend to form relationships based on shared attributes. Certain attributes that are ambiguous, stigmatized or illegal can create a ÔhiddenÕ population, so-called because its members are difficult to identify. Many hidden populations are also at an elevated risk of exposure to infectious diseases. Consequently, public health agencies are presently adopting modern survey techniques that traverse social networks in hidden populations by soliciting individuals to recruit their peers, *e.g.*, respondent-driven sampling (RDS). The concomitant accumulation of network-based epidemiological data, however, is rapidly outpacing the development of computational methods for analysis. Moreover, current analytical models rely on unrealistic assumptions, *e.g.*, that the traversal of social networks can be modeled by a Markov chain rather than a branching process.

**Methodology/Principal Findings:**

Here, we develop a new methodology based on stochastic context-free grammars (SCFGs), which are well-suited to modeling tree-like structure of the RDS recruitment process. We apply this methodology to an RDS case study of injection drug users (IDUs) in Tijuana, México, a hidden population at high risk of blood-borne and sexually-transmitted infections (*i.e.*, HIV, hepatitis C virus, syphilis). Survey data were encoded as text strings that were parsed using our custom implementation of the inside-outside algorithm in a publicly-available software package (HyPhy), which uses either expectation maximization or direct optimization methods and permits constraints on model parameters for hypothesis testing. We identified significant latent variability in the recruitment process that violates assumptions of Markov chain-based methods for RDS analysis: firstly, IDUs tended to emulate the recruitment behavior of their own recruiter; and secondly, the recruitment of like peers (homophily) was dependent on the number of recruits.

**Conclusions:**

SCFGs provide a rich probabilistic language that can articulate complex latent structure in survey data derived from the traversal of social networks. Such structure that has no representation in Markov chain-based models can interfere with the estimation of the composition of hidden populations if left unaccounted for, raising critical implications for the prevention and control of infectious disease epidemics.

## Introduction

Hidden populations consist of individuals sharing one or more common attributes that are masked from public surveillance, either because they are rare, difficult to measure or define (*e.g.*, jazz musicians [1]), or stigmatized and/or illegal (*e.g.*, injection drug use [2]). At the same time, many hidden populations are important foci for public health surveillance and outreach, *e.g.*, at greater risk of transmitted diseases. Social sampling techniques can overcome these obstacles by exploiting the social networks that permeate hidden populations. For instance, chain-referral sampling techniques such as ‘snowball’ sampling [3] solicit members of the hidden population to provide contact information on behalf of their peers. However, conjectures from chain-referral samples are susceptible to the non-randomness of the initial sample (‘seed’ individuals), which tends to comprise the most accessible members of the hidden population (*e.g.*, those enrolled into an institutional setting, such as a drug treatment program [4], [5]). In addition, members of a stigmatized or illegal hidden population may be reluctant to submit contact information without the consent of their peers. Respondent-driven sampling (RDS) is a recent offshoot of chain-referral sampling [6] that implements a dual-incentive system: in addition to an initial incentive for participation, a respondent receives additional rewards for every peer they recruit into the study. Respondents are given multiple study referral ‘coupons’ with which to recruit peers, which allows peers to decide for themselves whether or not to participate. An efficient and cost-effective sampling method, RDS is rapidly becoming the *de facto* standard for sampling from hidden populations [1], [7], [8] and is a critical component of the global effort against the AIDS epidemic [9].

A sample obtained using RDS provides detailed information about the social network, from which one can derive a less biased estimate of the composition of the hidden population [10], *e.g.*, what proportion are HIV-infected. This is possible because the propagation of referrals through a social network superficially resembles a first-order Markov chain that visits discrete states corresponding to various attributes of successive respondents, and converges to a stationary distribution independent of starting conditions. Consequently, social mixing (*i.e.*, non-random network associations) can be measured by cross-tabulating attributes of the recruiter against the recruitee, analyzed using log-linear models [11], and generalized to the population. However, this approach requires several inappropriate or oversimplifying assumptions. Firstly, the common practice of encouraging respondents to recruit multiple peers in order to accelerate the sampling process results in a referral ‘tree’ instead of a chain. Secondly, the recruitment process may be related not only to attributes of the recruiter, but the recruiter's recruiter also, and so on, leading to higher-order recruitment processes. Thirdly, the recruitment process may be influenced by unobserved states of respondents that might not be directly measurable, *e.g.*, an individual's disposition to recruit their peers.

**Figure 1:**
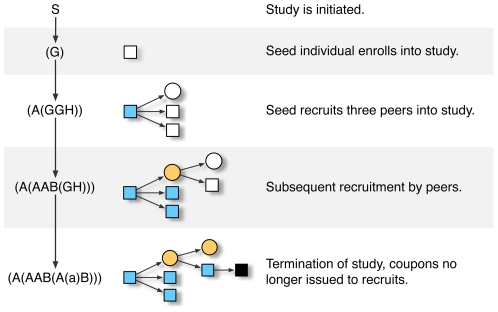
Relating a stochastic context-free grammar (SCFG) to the multitype branching process of RDS. Strings generated by an SCFG are depicted on the left-hand side. 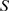 is the start symbol from which all strings are derived, corresponding to the initiation of an RDS study; 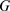 and 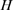 are non-terminal symbols for different types of respondents whose recruitment outcomes are unresolved; and 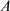 and 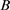 are terminal symbols for respondents whose recruitment outcomes are resolved. A successful recruitment is indicated by a substring enclosed in parentheses to the immediate right of the originating terminal symbol. To represent the termination of an RDS study, the remaining non-terminal symbols are rewritten as lower-case terminal symbols, *e.g.*, 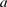.

Multitype branching processes (MBPs) provide a more natural representation of the tree-like structure of RDS recruitment process as it traverses the social network in a hidden population. Here, we exploit the close relationship between MBPs and stochastic context-free grammars (SCFGs) [12], [13] to develop a rich probabilistic language for the analysis of RDS data. A grammar comprises a set of rules for generating strings (*i.e.*, linear sequences of symbols) through the successive replacement of symbols until only non-replaceable (terminal) symbols remain [14]. Any tree-like structure can be encoded as a bracketed string, in which symbols are grouped by parentheses [15]; this principle is exemplified by the ubiquitous Newick format for phylogenetic trees, *e.g.*, “(( *human*, *chimpanzee*), *gorilla*)” [16]. Context-free grammars can generate strings only by replacing one non-terminal symbol at a time with one or more other symbols, and are therefore especially well-suited for modeling the growth of tree-like structures. In other words, we let the stochastic replacement of symbols in a string correspond to branching events in the recruitment process of RDS (Figure 1). Because a grammar may provide more than one derivation for a given string, we assign probabilities to different rules of the grammar that together define a joint probability for each derivation. As a result, stochastic grammars provide a robust means for inferring unobserved quantities from the data, *e.g.*, the dependence of recruitment dynamics on unmeasured latent variables. In sum, the versatile probabilistic framework of SCFGs enables us to perform rigorous hypothesis testing on RDS data for a wide range of models of recruitment dynamics.

To illustrate the application of SCFGs to the analysis of RDS data, we estimate recruitment dynamics and mixing patterns between discrete groups of individuals in a hidden population of injection drug users (IDUs) in Tijuana, México. Injection drug use is a growing problem in cities located along the U.S.-México border. México is the primary source of methamphetamine entering the United States [17] and a major source of cultivated heroin [18]. Cities and towns positioned on drug trafficking routes often experience epidemics of injection drug use [18], [19]. Tijuana is home to approximately 10,000 IDUs [18], who are at increased risk of infection with human immunodeficiency virus type 1 (HIV-1), hepatitis C virus (HCV), and sexually transmitted infections (STIs) such as syphilis. Elucidating the patterns of social mixing in this population is critical for recruitment of IDUs into surveillance and prevention studies aimed at monitoring and reducing the burden of these infectious diseases.

## Methods

### Ethics statement

Study methods were approved by the Institutional Review Board of the University of California, San Diego and the Ethics Board of the Tijuana General Hospital. Subjects gave their written informed consent to participate in the study.

### Study population

From February through April 2005, eligible IDUs were enrolled in a cross-sectional study in Tijuana, México. Eligibility criteria for the study included: having injected illicit drugs within the past month, confirmed by inspection of injection stigmata (*i.e.*, ‘track marks’); aged 18 years or older; willing and able to provide informed consent; and not having been previously interviewed for the study. Recruitment was performed by the Centro de Integración y Recuperación para Enfermos de Alcoholismo y Drogadicción “Mario Camacho Espíritu”, A.C. (CIRAD), a non-governmental organization (NGO) started in 1991 to work with drug users, who made weekly trips to three geographically diverse ‘colonias’ (*i.e.*, neighborhoods) in the city: Zona Norte, Camino Verde, and Sepanal, using a modified recreational vehicle that operated as a mobile clinic (the ‘Prevemovihl’).

Upon enrollment, trained staff administered quantitative surveys eliciting information on topics such as socioeconomic and demographic profiles, and drug use practices. Blood samples were obtained by venipuncture and serum was stored at the municipal health clinic in Tijuana before being shipped frozen to the San Diego County public health laboratory. Samples were tested for syphilis antibody with the rapid plasma reagin test (RPR Macrovue, Becton Dickinson) and, if reactive, confirmed by a *Treponema pallidum* particle agglutination assay (TPPA, Fujirebio Diagnostics). Each respondent received 10 U.S. dollars (USD) and, until the end of the study, three unique ‘coupons’ to refer their peers to the study. Respondents received an additional 5 USD for every eligible individual they recruited. Monetary incentives were determined based on the experience of study staff. Further details of the study are described in Frost *et al.* [11].

### Conversion of RDS data

Respondent data from the RDS study was deposited into a STATA (StataCorp, College Station, TX) database, including the unique identifiers of coupons issued to respondents, as well as the identifier of the coupon with which they were referred. We used custom scripts in R (R Foundation for Statistical Computing, Vienna, Austria) and Python to match coupons between recruiter and recruitee, and to encode the ensuing recruitment trees into strings (Supporting Text S2). Each string was comprised of the following terminal symbols: ‘ .’ to encode respondents irrespective of state; ‘ A’ and ‘ B’ to encode respondents with respect to observed attributes, *i.e.*, visible states; ‘ (‘ and ’)’ to encode nested groups within the tree-like structure of RDS (Figure 1). In addition, we made the replacements ‘ .’ 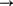 ‘ :’, ‘ A’ 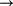 ‘ a’, and ‘ B’ 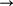 ‘ b’ to censor respondents that were not issued coupons near the end of the study from our model inference procedure. By censoring respondents without coupons, we avoid biasing our parameter estimates of recruitment behavior such as the probability of failing to recruit any peers. To reduce the complexity of the grammar, all substrings comprised of letters were pre-sorted in alphabetical order (equivalent to rotating branches of the recruitment tree), *e.g.*, “ A(ABA)” was probabilistically equivalent to “ A(AAB)”.

### Modeling RDS using SCFGs

Alternative models of the recruitment process were represented by SCFGs, each of which stipulated a different joint probability for a given string. A context-free grammar is comprised of rules that replace a single non-terminal symbol with a substring of non-terminal and terminal symbols. Rules in an SCFG may be weighted by different probabilities, which become the parameters of the model. The likelihood of an SCFG for a given set of strings was calculated by inferring the rule probabilities that were most likely to derive the corpus. In the absence of hidden states, these parameter estimates were equivalent to the frequency of each rule, *i.e.*, recruitment outcome. In order to capitalize on pre-existing efficient algorithms for inferring the most likely derivation of a string for a given grammar, all SCFGs were expressed in Chomsky normal form (CNF) such that a non-terminal symbol could be replaced only by either a pair of non-terminal symbols or a single terminal symbol [20]. Although this requirement may seem overly restrictive, in fact any context free grammar can be expressed in CNF [21]. For readability, we will present grammars in their non-CNF form. To analyze strings using SCFGs, we implemented the inside-outside [13] and Cocke-Kasami-Younger algorithms [22], [23] as a new component of the publicly-available software package *HyPhy* [24]. Confidence intervals around the maximum likelihood estimates of model parameters were obtained by likelihood profiling (Supporting Table S1).

We used the following notation to distinguish between different SCFG models in our study: 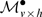, where 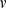 corresponds to the number of visible states, 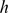 to the number of hidden states, and superscripts were used to label alternative parameterizations (*e.g.*, probability distributions) on the rule set defined by 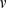 and 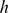. For example, the simplest SCFG model of the RDS process (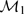) comprised the following rules and probabilities: 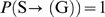; 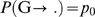; 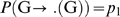; 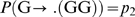; and 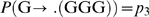. ‘S’ is the start symbol from which all strings are derived, and ‘G’ is a non-terminal symbol representing a potential recruitment, *i.e.*, an unredeemed coupon issued to a respondent. Each recruitment outcome per respondent (enclosed in parentheses and immediately following ‘ .’) is associated with a different probability with the constraint that 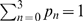, *i.e.*, the model has 3 free parameters. We will refer to 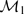 as a uniform recruitment model because we make no distinction among individuals with respect to visible attributes or hidden variation in recruitment behavior. Assuming the parameters 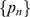 are known, the probability of generating the string “ (.(..))” is 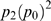. Conversely, the likelihood of 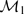 given this string is maximized by setting 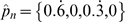 such that 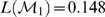. Constraining the 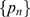 parameters to a binomial distribution such that 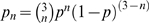 reduces the number of free parameters to 1. Because this is an alternative parameterization on the grammatical rules of 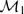, we denote this binomial model as 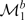. Its likelihood is maximized at 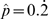 such that 
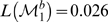
, almost six times lower than 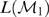. If we assume that the null distribution for the ratio of likelihoods is sufficiently approximated by a 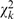 distribution, where 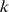 is the difference in the number of free parameters between models, then the probability that 
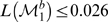
 is approximately 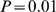. In other words, the removal of two parameters by constraining recruitment probabilities to a binomial distribution results in significant loss in model likelihood. This likelihood ratio test requires that the models are nested, *i.e.*, that one model can be defined in terms of the second. Relative performance of non-nested models was quantified by the difference between the 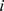-th model and the best model in terms of AIC, *i.e.*, 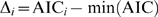. By convention, 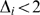 indicates substantial support for the 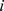-th model, which diminishes rapidly with increasing 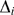 to a cut-off of 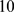, beyond which support is considered to be negligibly small [25].

In order to model variation in the recruitment process among respondents with respect to binary visible attributes, we employed an SCFG with the following rules and probabilities:

S 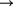 (G)
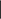
(H)
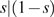


G 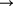 A
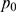


G 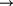 A(G)
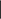
A(H)
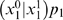


G 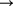 A(GG)
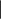
A(GH)|A(HH)
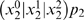


G 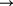 A(GGG)
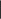
A(GGH)
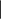
A(GHH)
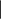
A(HHH)
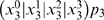


H 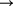 B
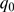


H 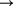 B(H)
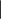
B(G)
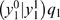


H 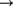 B(HH)
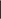
B(GH)
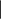
B(GG)
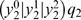


H 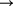 B(HHH)
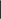
B(GHH)
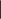
B(GGH)
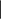
B(GGG)
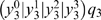


where ‘
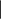
’ is used to separate outcomes, and 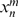 (or 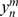) give the probability that 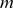 out of 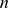 recruitees had the opposite state to a recruiter in state G (or H), such that 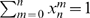 and 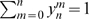 for 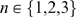. We also included the deterministic rules G 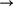 a and H 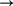 b to allow censored respondents to be interpreted as unresolved non-terminal symbols. This SCFG comprised 19 free parameters that could be constrained to express alternative models of recruitment. For instance, assuming a constant distribution in the number of recruits across visible states (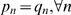) and binomial mixing probabilities (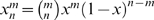) reduced the number of free parameters to 6. This parameterization of the SCFG was used as the baseline model, 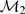. The complete set of SCFGs used in this study are described in Supporting Text S1.

## Results

### Variation in recruitment ability

**Figure 2:**
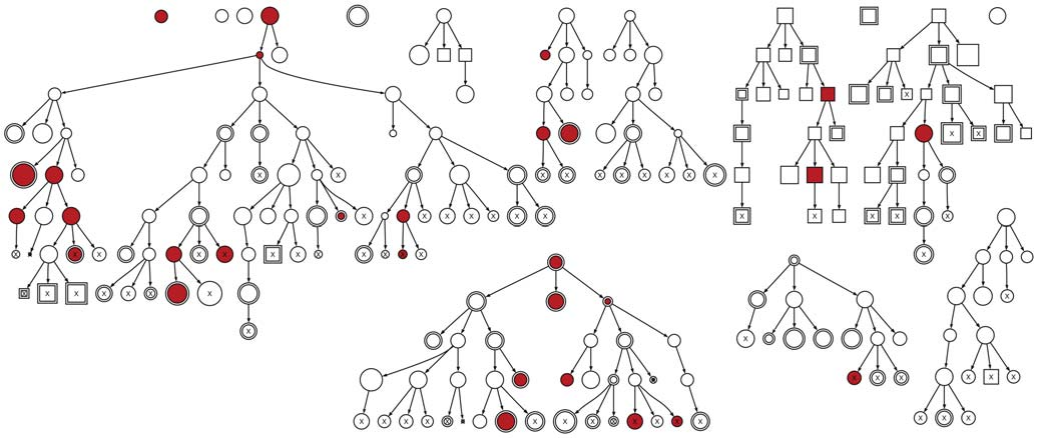
Referral trees from an RDS study of IDUs in Tijuana, México. Each node represents a respondent who recruits zero to three peers, indicated by arrows originating from the recruiter to the recruitee. The nodes are sized such that their diameter represents the respondent's reported network size on a logarithmic scale (base 10). A cross-mark inscribed within the node indicates that the respondent was not given coupons to recruit at the end of the study. Node shape indicates the interview site (circle = Sepanal/Zona Norte, square = Camino Verde). A doubled perimeter indicates that the respondent reported injecting drugs at home. Shaded nodes represent respondents who tested seropositive for syphilis.

**Figure 3:**
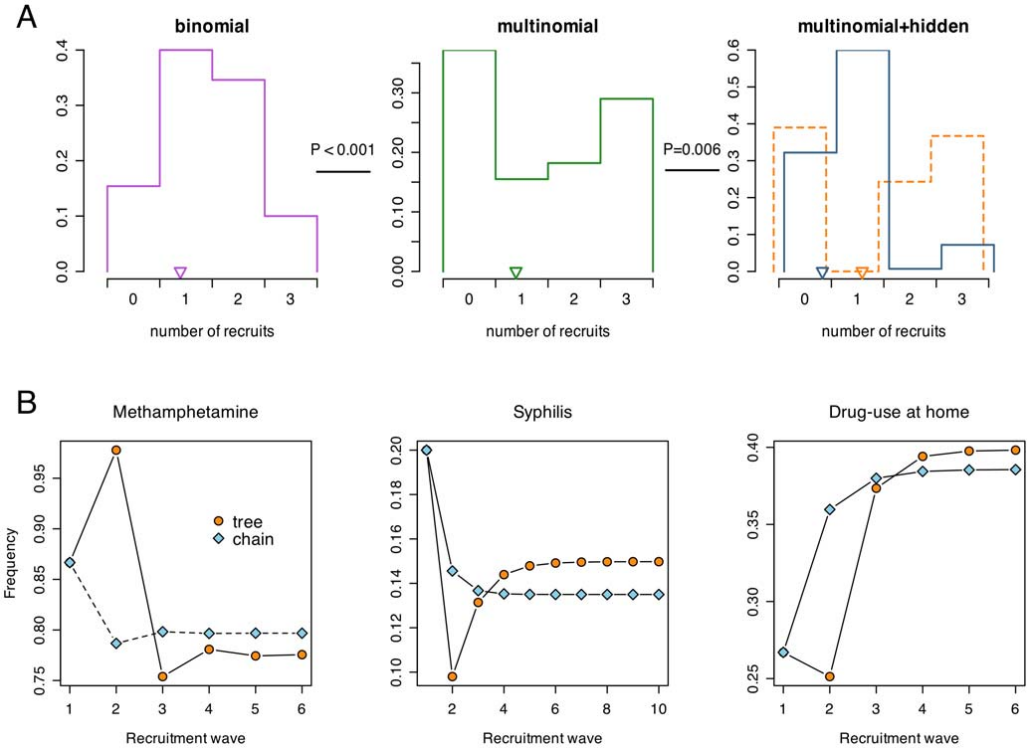
Contrasting model predictions of variation in recruitment dynamics. ( *A*) Histograms depict the inferred probability distribution for the number of recruits per respondent under the corresponding model. The dotted line histogram indicates the distribution in recruitment ability for respondents in the same hidden state as seed individuals. The mean number of recruits per respondent is indicated for each distribution by an inverted triangle along the x-axis. Plots are separated by *P*-values obtained from likelihood ratio tests between adjacent models (always favoring the right-hand side). ( *B*) Contrasting projected composition of samples based on Markov chain and multitype branching process (tree) models of RDS, in which the latter included hidden states to model variation among respondents not explained by the corresponding visible attribute (*viz.*, reporting prior use of methamphetamine, syphilis serostatus, and reporting injection drug use at home in the past six months). Frequencies correspond to the proportion of respondents in the ‘positive’ visible state (*i.e.*, using methamphetamines, syphilis seropositive, or using drugs at home).

The composition and recruitment dynamics of the respondents in this RDS study are depicted in Figure 2 as a ‘forest’ of 15 referral trees, with each tree rooted at a seed individual. Our use of SCFGs enabled us to analyze the dynamics of the recruitment process (*e.g.*, the number of recruits per respondent) by contrasting the likelihoods of different models of recruitment 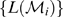, articulated by the assignment of probabilities to rules of the grammar. A conventional first-order Markov model of RDS implicitly assumes that the number of recruits follows a binomial distribution, *i.e.*, the outcome of three independent attempts to recruit with a constant probability of success. However, the likelihood of the equivalent SCFG with binomial recruitment numbers (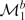) was significantly lower than an SCFG with multinomial recruitment numbers (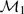), in which the only constraint on the probabilities of recruiting 0, 1, 2, or 3 individuals is summing to unity (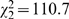, 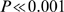). This outcome was due to the bimodal nature of the distribution of the number of recruits (Figure 3A), suggesting that the probability of recruitment was conditional on prior successes and/or that the propensity to recruit varied among respondents [11]. Because 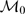 underestimated variation in the number of recruits per respondent, it also performed poorly relative to 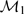 in predicting the distribution of referral tree sizes (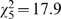, 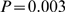; Supporting Figure S1).

We modeled variation in recruitment ability among respondents by expanding the SCFG to include non-observed or unobservable (‘hidden’) states of respondents, akin to a hidden Markov model operating within a branching process. Hidden states can represent different modes of recruitment, which could be interpreted as either an individual-level characteristic transmitted from recruiter to recruitee, or a population-level effect, *e.g.*, the recruitment process enters an unmeasurable ‘neighborhood’ within the social network in which recruitment dynamics are skewed. We denote the inclusion of hidden states into the multinomial uniform recruitment model by the notation 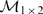, where the subscript indicates that respondents share a single visible state but divided into two hidden states. 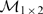 significantly improved likelihood relative to 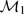 (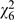 = 17.9, 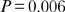 = 0.006) due to a positive correlation between the recruitment abilities of the recruiter and the recruitee (odds ratio, 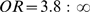). By computing the most likely derivation of the ‘observed’ strings under model 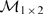 [22], [23], we mapped transitions between hidden states to specific recruitment events in the referral trees (Supporting Figure S2). All seed individuals were inferred to belong to the same ‘boom-or-bust’ hidden state, in which respondents recruited 1.6 peers on average despite a high attrition rate (Figure 3A). Recruitment processes subsequently evolved into an absorbing hidden state at a rate of 0.21 per recruitment, characterized by a more consistent but unsustainable rate of recruitment (averaging 0.83 recruits per respondent; Figure 3A). (A complete listing of parameter estimates for all SCFGs employed in this study is provided as Supporting Table S1.)

### Social mixing between visible states

To evaluate the extent of social mixing, we re-encoded the RDS data into strings using two different symbols (‘ A’, ‘ B’) to annotate binary visible states of respondents, *viz.*: syphilis serostatus; interview site, a proxy for geographic mixing; status as an active drug dealer or operator of a ‘shooting gallery’, a shared site for injection drug-use known as a ‘picadero’; whether the respondent injected drugs predominantly at home or in a shooting gallery; and reported use of methamphetamine. These strings were analyzed using an expanded SCFG model (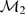) that accommodated binary visible states. In order to minimize the number of model parameters, we assumed initially that the probability of recruiting outside of one's visible category was independent of the number of recruits. The ‘full’ parameterization of 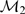 therefore comprised 9 degrees of freedom: 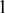 for the binomial distribution of visible states in seed individuals, 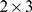 for the multinomial distributions in the number of recruits per visible state, and 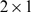 for the binomial distributions in visible states of recruitees per visible state of the recruiter. However, we found that for every visible state in our study, constraining the number of recruits per recruiter to be independent of the recruiter's visible state achieved a similar fit to the data with fewer parameters (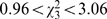, 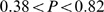). Subsequent extensions of 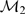 were therefore evaluated against this baseline assumption in the number of recruits except where noted otherwise.

The degree of assortative mixing, or homophily, with respect to a visible state was evaluated by computing the likelihood of an alternative model (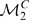) where the probability of recruiting peers with a given visible state was constrained to be independent of the recruiter's visible state. Due to the high rate of transit of IDUs along the Tijuana River canal between the Sepanal and Zona Norte interview sites, we merged respondents that were interviewed in these neighborhoods into a single location, SZN, leaving Camino Verde (CV) as the second location. We detected significant assortative mixing with respect to location (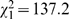, 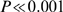). Recruitees tended to be interviewed at the recruiter's interview site more often, with probability 0.96 (95% CI: 0.92, 0.98) at SVM and 0.95 (95% CI: 0.85, 0.99) at CV. We also detected significant assortative mixing by syphilis serostatus (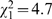, 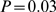). 31 out of 221 respondents (14%) tested seropositive for syphilis. However, a seropositive individual recruited seropositive peers with a probability of 0.28 (95% CI: 0.14, 0.45). In contrast, seronegative individuals recruited seropositive individuals with a probability of 0.11 (95% CI: 0.07, 0.16). Although syphilis serostatus is visible in statistical terms, it is likely to be invisible with respect to social mixing; consequently, assortative mixing between seropositive individuals is probably due to mixing with respect to other attributes related to the risk of STI acquisition.

Twenty-five respondents (11%) were categorized as ‘dealers’ if they had either sold drugs to IDUs within the past six months, or operated a shooting gallery. Our *a priori* expectation was that dealers would tend to recruit outside of their group, because forming ties with non-dealers may be essential to assuming this role in the hidden population, and that they would also be more effective recruiters. However, we found no evidence of either disassortative or assortative mixing (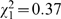, 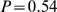); dealer or non-dealer respondents recruited dealer peers in roughly equal proportions. Additionally, 77 out of 221 respondents (35%) reported injection drug use at home within the past six months. We found significant assortative mixing among respondents with respect to reporting injection drug use at home (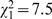, 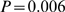). A disproportionate number of the female respondents (12 of 19) were injecting drugs at home relative to males (65 of 202; 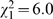, 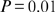 = 0.01) and may have frequently recruited their partners. Finally, 177 respondents (80%) reported use of methamphetamine, of whom 106 reported use within the six months prior to the interview date. We detected significant disassortative mixing with respect to methamphetamine use (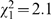, 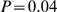). Non-user respondents recruited methamphetamine-using peers significantly more often than methamphetamine users, with respective probabilities 0.91 (95% CI: 0.79, 0.98) and 0.77 (95% CI: 0.70, 0.83).

Because the preceding analyses assume that mixing rates were independent of the number of recruits per respondent, similar results may be obtained using contingency table-based analyses that are often employed in the standard Markov chain treatment of RDS data. To evaluate the validity of this assumption, we modified the baseline 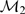 model so that the probability of a respondent recruiting outside of his/her visible group was dependent on the number of peers being recruited (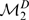). We found a significant improvement in likelihood for 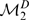 relative to 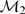 with respect to mixing by interview site (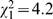, 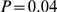). For instance, peers recruited by respondents interviewed at SZN were 3.7 (95% CI: 2.4, 5.2) times more likely to be interviewed at the CV site for every additional peer recruited. This provides our second example of recruitment dynamics that cannot be expressed by a first-order Markov chain.

### Combining hidden and visible states

So far we have assumed that social mixing was entirely dependent on visible states of respondents. However, it is impossible to measure exhaustively the characteristics of respondents, especially in the context of hidden populations where potential respondents are less likely to participate in an over-thorough interview process, and other characteristics may be immeasurable (*i.e.*, latent variables). For example, apparent social mixing on a visible state may be confounded by real social mixing on a latent variable (*e.g.*, extroversion) when the two are correlated in the population. One of the advantages of SCFGs over conventional methods (*e.g.*, contingency tables) in the context of RDS data analysis is that one can extrapolate the influence of unmeasured or latent variation on the recruitment process. Here we explore this aspect of SCFG-based inference, firstly by evaluating whether mixing on visible states in our data set is confounded by mixing in latent space; and secondly by permitting mixing to be dependent on both visible and hidden states, such that mixing becomes a dynamic component of the recruitment process.

To determine whether our detection of mixing on visible states might be confounded by mixing on one or more latent variables, we evaluated an SCFG model (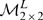) in which recruitment of like peers was independent of the recruiter's visible state, but dependent on a hidden state of the recruiter (see Supporting ). This ‘latent-mixing’ model was analogous to populating two ‘islands’ in latent space with divergent frequencies of a visible state and restricting migration between islands. We employed the delta-AIC (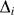) statistic [25] to compare non-nested models 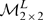 and 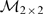. In the case of interview site as visible state, we found that 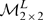 performed extremely poorly (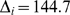). Moreover, the likelihood of 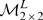 for interview site was very close to that of model in which mixing was entirely independent of any state of the recruiter. These results indicated that apparent mixing with respect to interview site was very unlikely to be explained by mixing on latent variables, *i.e.*, interview site was an effective proxy for one or more ‘socially visible’ characteristics.

In contrast, the 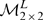 model performed nearly as well or better than 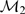 for the remaining visible states. With respect to syphilis serostatus, a value of 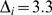 indicated that mixing on latent variables could provide a reasonable approximation to genuine mixing on this visible state. Put another way, it is unreasonable to assume that respondents were aware of their peers' syphilis serostatus, whereas incidental mixing on this state could have occurred due to mixing on latent variables such as sexual transmission risk behavior or geographic clustering. Mixing with respect to drug-dealing status was less satisfactorily explained by the 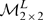 model (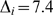), implying that respondents were relatively more aware of whether their peers engaged in drug dealing or operating a ‘shooting gallery’. On the other hand, apparent mixing with respect to the use of drugs at home may have been confounded entirely with mixing on one or more latent variables (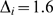), suggesting that this behavior was not socially visible. Methamphetamine use was unique amongst all visible states in that the 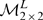 model attained a lower AIC value than the baseline 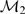 model. Parameter estimates in the 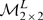 model indicated that seed individuals were more likely to recruit peers that did not use methamphetamine, regardless of whether the recruiters used this drug. Methamphetamine users tended to appear in later recruitment waves, manifested in the 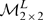 model by transitions in latent space.

Results from our analysis of RDS data using the 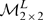 model implied a general lack of evidence supporting the assumption that apparent mixing on visible states was entirely independent of the visible state of the recruiter. To relax this assumption while retaining hidden variation in mixing probabilities, we investigated a more complex model, denoted as 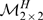. On the basis of results from the 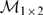 model, we assigned independent multinomial distributions to each hidden state, *i.e.*, to permit transitions over time in the distribution of the number of recruits per respondent. In addition, we assumed that the probabilities of recruiting peers with respect to visible and hidden states was dependent on both visible and hidden states of the recruiter. Under this set of assumptions, the 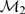 model could be defined as a special case of the 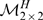 model, *i.e.*, the models were nested. We found a significant improvement in likelihood for this extension of the model with respect to methamphetamine use (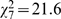, 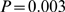), syphilis serostatus (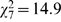, 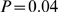), and reporting injection drug use at home (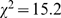, 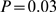; Supporting Table S1). As expected, we observed the same ‘boom-or-bust’ dynamics characteristic of early recruitment events in the study, but we also detected hidden variation in mixing. As noted in our analysis of the latent-mixing model (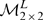), early respondents reporting use of methamphetamine displayed stronger associative mixing (recruiting ‘like’ with probability 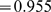, 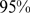 CI: 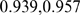) than did similar respondents at a later stage of the study (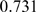, 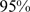 CI: 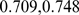). Moreover, the 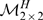 model obtained a lower AIC value when analyzing methamphetamine use than did 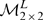 (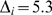).

Hidden variation in the recruitment process can confound estimates of the numbers of seeds and numbers of waves required to obtain a given sample size, or the projected composition of the sample over successive recruitment ‘waves’. To illustrate, we compared the predictions of the standard first-order Markov chain approach to the best-fitting SCFG with respect to methamphetamine use. The 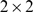 contingency table for 206 recruitment events was:
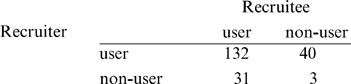
from which transition probabilities of the Markov chain were obtained by normalizing cell counts by the row totals [6]. As expected, the resulting matrix was identical to the mixing probabilities estimated from an SCFG modeling two visible states without hidden variation. From the more likely SCFG including both visible and hidden states, we obtained a matrix of first moments [26] for the MBP represented by this grammar:
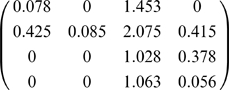
which acts on the probability vector of states 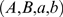, in which 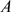 and 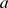 represent the initial and derived hidden states within the methamphetamine-user subpopulation, respectively; and 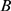 and 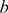 represent the corresponding hidden states for non-users.

The transient dynamics of the recruitment processes under both models are illustrated in Figure 3B for all three visible attributes. In the case of mixing with respect to methamphetamine use, there is a sharp discrepancy between model predictions after one recruitment wave, due to the idiosyncratic recruitment behavior in seed individuals that was captured by the addition of hidden states. Failing to account for hidden variation in the recruitment process skews the estimates of mixing probabilities, which is illustrated by divergent equilibrium frequencies after multiple recruitment waves. For instance, syphilis seropositive respondents initially recruited with complete homophily, which then regressed to a more random recruitment behavior (Supporting Table S1).

## Discussion

RDS is rapidly becoming the *de facto* standard to recruit hidden populations, *e.g.*, jazz musicians [1] and drug users in New York [7]–[9]; artists in Philadelphia [27]; MDMA (3,4-methylenedioxy-N-methylamphetamine) users in Ohio [28]; Latino gay and bisexual men, and transgender (male-to-female) persons in Chicago and San Francisco [29]; sex workers in Eastern Europe [30]; and injection drug users in Mexico [11], Russia, and Estonia [31]. The Centers for Disease Control and Prevention (CDC) is employing RDS for its National HIV Behavioral Surveillance (NHBS) to recruit injection drug users (IDUs), men who have sex with men (MSM), and high risk heterosexuals in 25 U.S. cities [32]; IDUs in Bangkok; and IDUs and commercial sex workers in Vietnam [33], as part of its Global AIDS Program [9]. In addition, Family Health International (FHI) is using RDS to study MSM, IDUs, and commercial sex workers in over a dozen countries, including Bangladesh, Myanmar, Cambodia, Egypt, Honduras, India, Indonesia, Mexico, Nepal, Vietnam, Pakistan, Papua New Guinea [34], Albania, and Russia [35], [36]. Thus, there will soon be a tremendous number of data sets based on this sampling method requiring accurate and informative methods of analysis.

SCFGs provide a versatile framework for hypothesis testing on RDS data using likelihood-based methods of model selection, *e.g.*, likelihood ratio test or Akaike's information criterion (AIC). Our custom software implementation of SCFGs enables the user to: (*i*) evaluate the likelihood of any MBP model of recruitment that can be expressed in grammatical form; (*ii*) infer unobserved quantities using hidden states; (*iii*) employ any combination of constraints on model parameters; (*iv*) obtain maximum likelihood parameter estimates with confidence intervals derived from likelihood profiles, and; (*v*) censor late respondents who were not issued referral coupons. This software is freely available (http://www.hyphy.org) and represents an important resource for RDS and other applications of natural language processing.

Hidden variability in recruitment ability may explain the ‘boom-or-bust’ nature of RDS. For example, in a survey of RDS feasibility studies to recruit from sex worker populations in Eastern Europe, Simic *et al.* [30] found that no more than half of seed individuals recruited any peers, despite indicating a willingness to participate in the RDS study and a substantial network size (5–20 peers). None of these studies reached their target sample size and nearly all respondents were recruited in productive chains descended from one or two seeds. Hence, the expected number of recruits per respondent is a poor predictor of the sample size or number of recruitment waves produced from a given number of seed individuals. In our case study, the overall mean number of recruits was 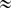
1.4 per respondent. Based on this information alone, *i.e.*, assuming a binomial recruitment model, our simulations predict that 71% of RDS studies initiated from a single seed individual would successfully obtain a target sample size of 500 respondents. Under the multinomial model of recruitment, this success rate declines to 45% using parameter estimates from our case study, and even further yet with the addition of hidden states (30%). In sum, our best-fitting uniform recruitment model indicates that an RDS study would require at least three times as many seed individuals to obtain the same yield predicted by the Markov chain model of RDS. More importantly, this target yield is accomplished in less than half as many recruitment waves. In other words, the ‘boom-or-bust’ dynamics of RDS as revealed by SCFGs may prevent RDS from penetrating a hidden population.

Although the MBP-based models of RDS that we espouse here allay unrealistic assumptions of first-order Markov chain models, both conventional and MBP-based models of RDS make independence assumptions about recruitment dynamics. Firstly, we assume that the social network provides a constant supply of potential recruits, independent of the recruitment process. This assumption is breached when a social network exhibits strong transitivity (‘the friend of my friend is also my friend’) such that respondents may attempt to recruit peers sampled in previous recruitment waves. Secondly, if seed individuals occupy similar positions in the network, then a greater chance exists that their recruitment trees will collide, *i.e.*, that multiple respondents will attempt to recruit the same peer. The similarity of RDS to the classic susceptible-infected-recovered (SIR) epidemiological model, in which individuals become ‘infectious’ upon recruitment and can infect only a diminishing number of peers, may offer some recourse for addressing this effect. However, current techniques for estimating parameters of stochastic SIR models assume either panmixis (a fully-connected social network) or simple population structures, such as households.

Something of interest is the possibility that respondents recruit non-randomly from their personal networks. However, this does not breach any assumption of our model; rather, non-random recruitment affects the interpretation of RDS data on the whole, *i.e.*, estimating frequencies of attributes in a hidden population. The extent of non-random recruitment may be determined by interviewing respondents on the composition of their personal networks and comparing their estimates to recruitment outcomes. Indeed, by using this assessment previous investigators have found that respondents in their respective RDS studies were evidently recruiting at random from their personal networks [28], [37]. Respondent estimates of their personal networks could also be used to weight discrepant RDS outcomes (but see below).

We have focused on the ability of SCFGs, as an implementation of MBPs, to reveal important information about mixing patterns in hidden populations from RDS data. RDS is also used to estimate the composition of hidden populations by the application of weights, similar to other adaptive sampling designs [38]; likewise, SCFGs (in which rules for generating strings are weighted by probabilities) can accommodate additional weights. However, survey weighting is exceedingly difficult [39] and the generation of appropriate weighting schemes (particularly in the presence of hidden variables) is a challenging area for further research in RDS. The network sizes collected in order to generate these weights also provide information on mixing of different populations [40]. In addition, despite being restricted to modeling tree-like structures, one can gather information about transitivity through questions such as “*Do your recruits know your recruiter?*”, which can be considered as an additional state. With the generation of many large and complex RDS data sets, the further development of analytical methods will require collaborative work spanning several disciplines, before we can fully appreciate the social determinants of susceptibility to infectious disease epidemics in human populations.

## Supporting Information

Figure S1 Model predictions of RDS network component size distribution. The observed RDS network components, or referral trees, were ranked according to size (number of respondents; solid circles). Predicted distributions of ranked component sizes were obtained using maximum likelihood estimates of model parameters for the binomial (crosses), multinomial (open triangles), and multinomial + hidden (plus signs) recruitment models, displayed on the same plot for comparison. We find that the binomial model severely underestimated the size of the largest-ranked components, due to its inflexibility in modeling variation in recruitment among respondents.
(0.21 MB TIF)

Figure S2 Cocke-Kasami-Younger reconstruction of hidden states in recruitment dynamics. All seed individuals in the RDS study were grouped into a shared hidden state with respect to recruitment dynamics (open circles), characterized by ‘boom-or-bust’ recruitment with a relatively high mean number of recruits. Hidden states were ‘transmitted’ from recruiter to recruitee in an autocorrelated fashion until a switch occurred into a second hidden state (filled circles), characterized by more consistent recruitment of fewer peers.
(0.51 MB TIF)

Text S1 (0.07 MB PDF)

Text S2 (0.04 MB PDF)

Table S1 Model parameter estimates are grouped by ‘visible’ attributes of respondents (location, syphilis serostatus, drug-dealing, drug-use at home, and use of methamphetamine). Each model of recruitment was evaluated using a custom SCFG, comprising the rules listed with each hypothesis: *2-visible*, full recruitment model with binary visible states; *constrain mix*, constrained mixing rates between visible states to be equal; *constrain N* (M_2_ baseline model), constrained distribution in the number of recruits per respondent to be equal; *constrain both* (M_2_^C^), constrained both mixing rates and distributions in the number of recruits; (5) *dependent mixing* (M_2_^D^), permitted mixing rates to be dependent on the number of recruits; (6) *latent-mixing* (M_2×2_^L^), mixing on latent variables, independent of the visible state of the recruiter; and (7) *add hidden* (M_2×2_^H^), addition of hidden states representing variation in mixing rates and number of recruits over time. Production rules for each grammar are presented in non-reduced form to conserve space. Column header abbreviations are defined as follows: MLE = maximum likelihood estimate of model parameter; *lower, upper 95%* = lower and upper limits of the 95% confidence interval, estimated using likelihood profiling; *log L*, log-transformed likelihood of the model; *df* = degrees of freedom, *i.e.*, number of free parameters in the model; AIC = Akaike's Information Criterion.
(0.09 MB PDF)
