# Supplementary figures and images for: Parsing Social Network Survey Data from Hidden Populations Using Stochastic Context-Free Grammars
Source: PLoS One. 2009 Sep 7;4(9):e6777. doi: 10.1371/journal.pone.0006777 (PMC2734164; doi:10.1371/journal.pone.0006777)

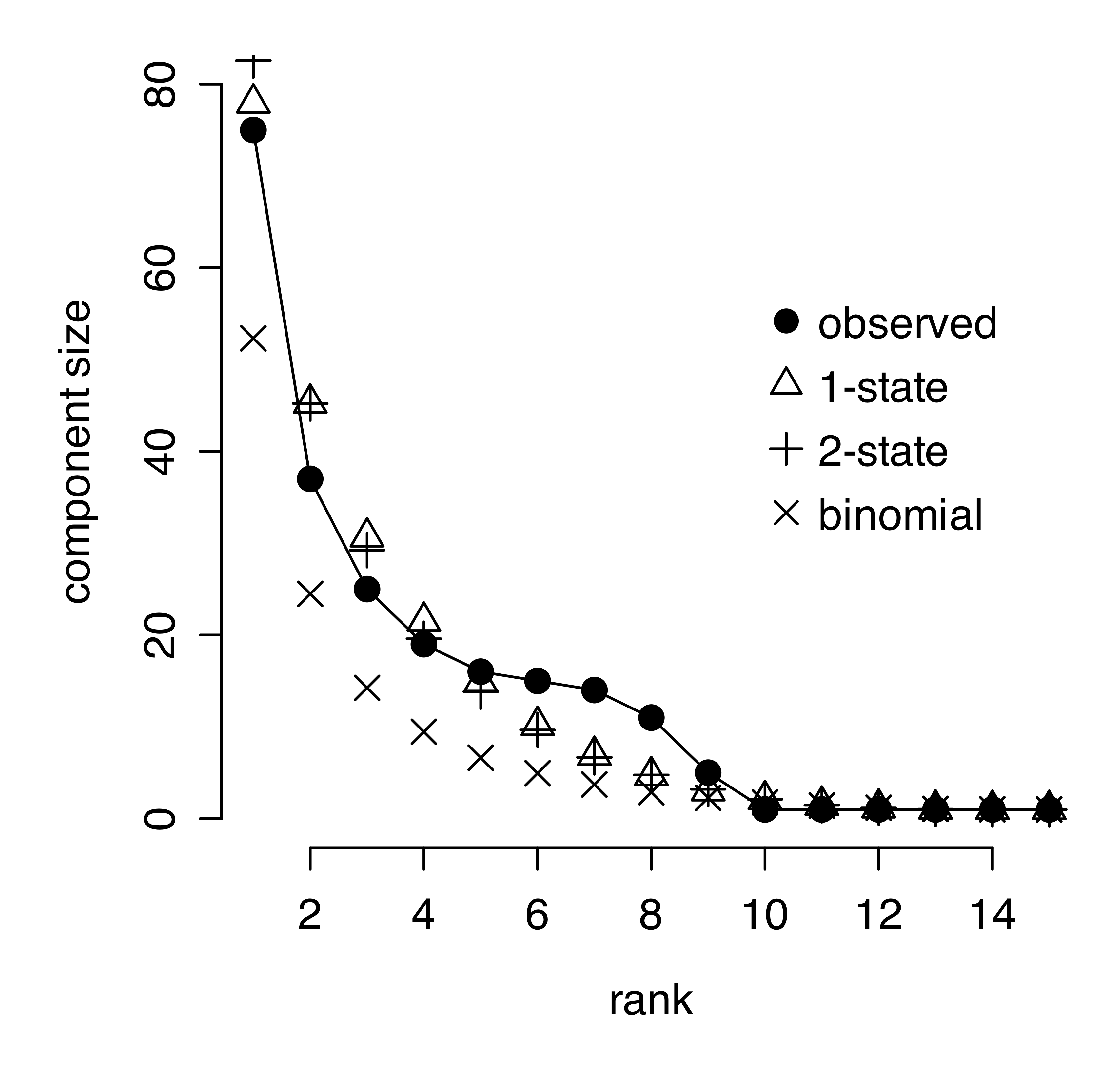

Supplement: Figure S1 — Model predictions of RDS network component size distribution. The observed RDS network components, or referral trees, were ranked according to size (number of respondents; solid circles). Predicted distributions of ranked component sizes were obtained using maximum likelihood estimates of model parameters for the binomial (crosses), multinomial (open triangles), and multinomial + hidden (plus signs) recruitment models, displayed on the same plot for comparison. We find that the binomial model severely underestimated the size of the largest-ranked components, due to its inflexibility in modeling variation in recruitment among respondents. (0.21 MB TIF) [file pone.0006777.s001.tif]

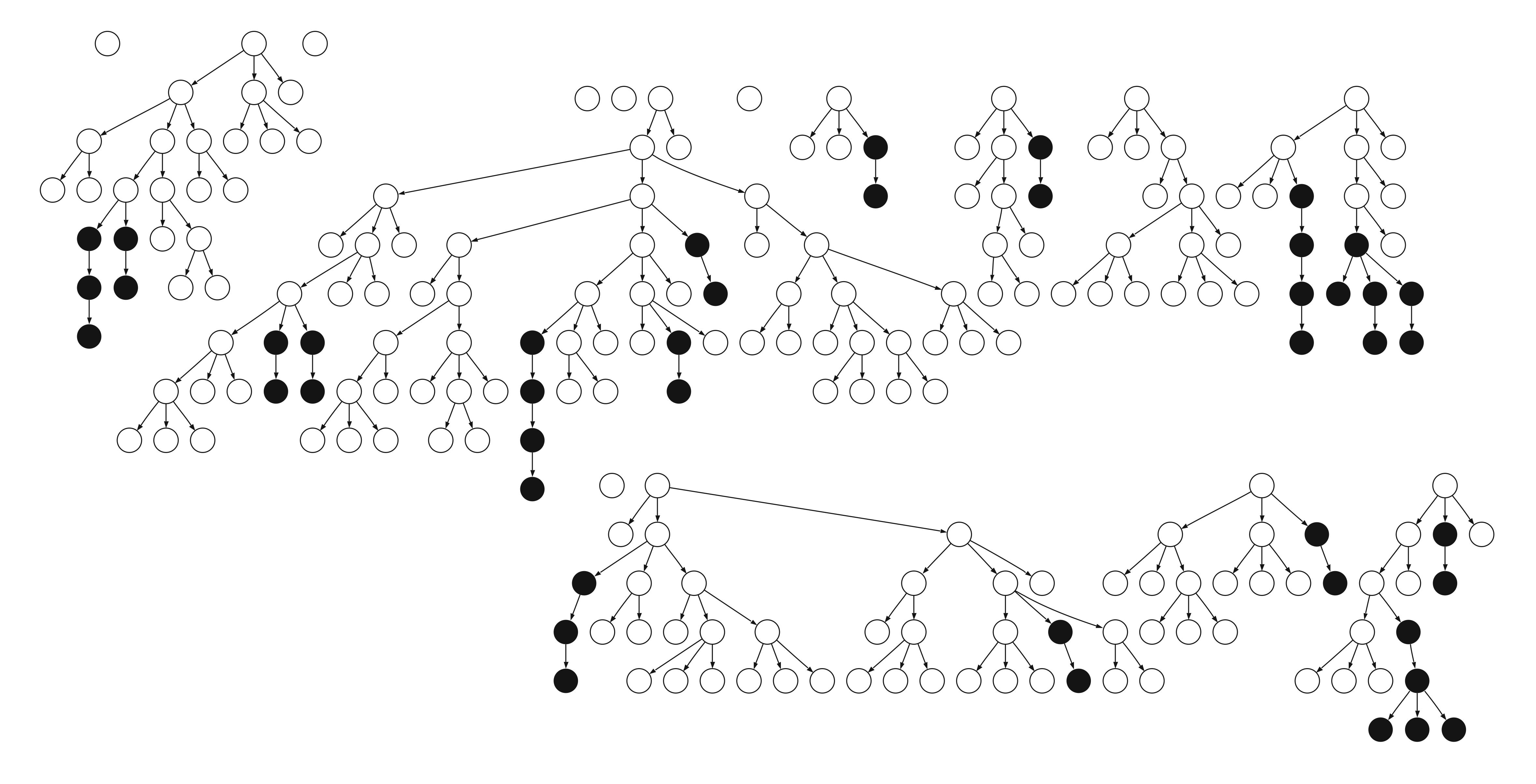

Supplement: Figure S2 — Cocke-Kasami-Younger reconstruction of hidden states in recruitment dynamics. All seed individuals in the RDS study were grouped into a shared hidden state with respect to recruitment dynamics (open circles), characterized by ‘boom-or-bust’ recruitment with a relatively high mean number of recruits. Hidden states were ‘transmitted’ from recruiter to recruitee in an autocorrelated fashion until a switch occurred into a second hidden state (filled circles), characterized by more consistent recruitment of fewer peers. (0.51 MB TIF) [file pone.0006777.s002.tif]
