# Supplementary material for: Parsing Social Network Survey Data from Hidden Populations Using Stochastic Context-Free Grammars
Source: PLoS One. 2009 Sep 7;4(9):e6777. doi: 10.1371/journal.pone.0006777 (PMC2734164; doi:10.1371/journal.pone.0006777)
Supplement: Text S1 — (0.07 MB PDF) [file pone.0006777.s003.pdf]

Supporting Text S1

Stochastic context-free grammars (SCFGs) employed in study. SCFGs are provided in Chomsky normal form. Different models of recruitment using the same grammar are defined by alternative parameterizations, *i.e.*, assignment of rule probabilities.

1. Uniform recruitment models:

Rule	Multinomial ($\mathcal{M}_1$)	Binomial ($\mathcal{M}_1^b$)
`S` → `bR`	1	
`b` → `(`	1	
`R` → `Gr`	1	
`r` → `)`	1	
`G` → `p`	p_1	p_1^3
`p` → `.`	1	
`G` → `pI`	p_2	$3p_1^2(1-p_1)$
`I` → `bJ`	1	
`J` → `Gr`	1	
`G` → `pV`	p_3	$3p_1(1-p_1)^2$
`V` → `bU`	1	
`U` → `GJ`	1	
`G` → `pW`	$1-p_1-p_2-p_3$	$(1-p_1)^3$
`W` → `bX`	1	
`X` → `GU`	1	
`G` → `:`	(1)	

Each model parameter is denoted by p_i where $max(i)$ corresponds to the degrees of freedom in the model. Note that the probabilities of rules for replacing the non-terminal symbol G sum to greater than unity because of an additional rule for censoring respondents (each encoded in the RDS string as '`:`'); this rule is disregarded during model inference. Chomsky normal form (CNF) of a grammar can be interpreted as an expansion of rules from their original form, as demonstrated in the following example: `G` → `pI` → `.bJ` → `.(Gr` → `.(G)`

2. Hidden state recruitment model ($\mathcal{M}_{1\times2}$), no visible differentiation among respondents:

Rule		Rule	
S → bR	1		
b → (	1		
R → Gr	p_1		
R → Hr	$1 - p_1$		
r →)	1		
G → d	p_2	H → d	p_6
d → .	1		
G → pI	p_3	H → pi	p_7
I → bJ	1	i → pj	1
J → Gr	p_4	j → Gr	p_8
J → Hr	$1 - p_4$	j → Hr	$1 - p_8$
G → pV	p_5	H → pv	p_9
V → bU	1	v → bu	1
U → GJ	p_4	u → Gj	p_8
U → HJ	$1 - p_4$	u → Hj	$1 - p_8$
G → pW	$1 - p_2 - p_3 - p_5$	H → pw	$1 - p_6 - p_7 - p_9$
W → bX	1	w → bx	1
X → GU	p_4	x → Gu	p_8
X → HU	$1 - p_4$	x → Hu	$1 - p_8$
G → :	(1)	H → :	(1)

Entries are left blank to indicate that the rule assumes the same probability parameterization as the preceding rule on the same row.

3. Binary visible state recruitment models ($\mathcal{M}_2$ family):

Rule	Full model	Constrain number of recruits ($\mathcal{M}_2$)	Constrain mixing rates ($\mathcal{M}_2^C$)	Mixing dependent on recruitment ($\mathcal{M}_2^D$)
`S` $\rightarrow$ `bR`	1			
`b` $\rightarrow$ `(`	1			
`R` $\rightarrow$ `Gr`	p_1			
`R` $\rightarrow$ `Hr`	$1 - p_1$			
`r` $\rightarrow$ `)`	1			
`G` $\rightarrow$ `d`	p_2			
`d` $\rightarrow$ `A`	1			
`G` $\rightarrow$ `dI`	p_3			
`I` $\rightarrow$ `bJ`	1			
`J` $\rightarrow$ `Gr`	p_4			
`J` $\rightarrow$ `Hr`	$1 - p_4$			
`G` $\rightarrow$ `dV`	p_5			
`V` $\rightarrow$ `bU`	1			
`U` $\rightarrow$ `GJ`	p_4			$p_4 p_7/(p_4 p_7 + 1 - p_4)$
`U` $\rightarrow$ `HJ`	$1 - p_4$			$(1 - p_4)/(p_4 p_7 + 1 - p_4)$
`G` $\rightarrow$ `dW`	$1 - p_2 - p_3 - p_5$			
`W` $\rightarrow$ `bX`	1			
`X` $\rightarrow$ `GU`	p_4			$p_4 p_7^2/(p_4 p_7^2 + 1 - p_4)$
`X` $\rightarrow$ `HU`	$1 - p_4$			$(1 - p_4)/(p_4 p_7^2 + 1 - p_4)$
`G` $\rightarrow$ `:`	(1)			
`H` $\rightarrow$ `e`	p_6	p_2	p_6	p_2
`e` $\rightarrow$ `B`	1			
`H` $\rightarrow$ `ei`	p_7	p_3	p_7	p_3
`i` $\rightarrow$ `bj`	1			
`j` $\rightarrow$ `Gr`	p_8	p_6	p_4	p_6
`j` $\rightarrow$ `Hr`	$1 - p_8$	$1 - p_6$	p_4	$1 - p_6$
`H` $\rightarrow$ `ev`	p_9	p_5	p_8	p_5
`v` $\rightarrow$ `bu`	1			
`u` $\rightarrow$ `Gj`	p_8	p_6	p_4	$p_6/(1 - p_6 p_7 + p_6)$
`u` $\rightarrow$ `Hj`	$1 - p_8$	$1 - p_6$	$1 - p_4$	$(1 - p_6)p_7/(1 - p_6 p_7 + p_6)$
`H` $\rightarrow$ `ew`	$1 - p_6 - p_7 - p_9$	$1 - p_2 - p_3 - p_5$	$1 - p_6 - p_7 - p_8$	$1 - p_2 - p_3 - p_5$
`w` $\rightarrow$ `bx`	1			
`x` $\rightarrow$ `Gu`	p_8	p_6	p_4	$p_6/(1 - p_6 p_7^2 + p_6)$
`x` $\rightarrow$ `Hu`	$1 - p_8$	$1 - p_6$	$1 - p_4$	$(1 - p_6)p_7^2/(1 - p_6 p_7^2 + p_6)$
`H` $\rightarrow$ `:`	(1)			

In constraining the number of recruits, we assume that the number of peers recruited by a respondent follows the same distribution regardless of the respondent's visible state. Similarly, in constraining the mixing rates, we assume that the probability that a respondent recruits peers in a given visible state is independent of the respondent's visible state.

4. Binary visible state model with mixing on latent variables ($\mathcal{M}^L_{2\times 2}$) or hidden states ($\mathcal{M}^H_{2\times 2}$), and constrained numbers of recruits:

Rule	Latent mixing	Hidden	Rule	Latent mixing	Hidden
S → bR	1				
b → (	1				
R → Gr	p_1	p_1			
R → Hr	p_2	$1-p_1$			
R → gr	p_3	0			
R → hr	$1-p_1-p_2-p_3$	0			
r →)	1				
G → d	p_4	p_2	g → d	p_4	p_9
d → A	1				
G → dI	p_5	p_3	g → dI'	p_5	p_{10}
I → bJ	1		I' → bJ'	1	
J → Gr	p_6p_7	p_4p_5	J' → Gr	$(1-p_9)p_7$	0
J → Hr	$p_6(1-p_7)$	$p_4(1-p_5)$	J' → Hr	$(1-p_9)(1-p_7)$	0
J → gr	$(1-p_6)p_8$	$(1-p_4)p_5$	J' → gr	p_9p_8	p_{11}
J → hr	$(1-p_6)(1-p_8)$	$(1-p_4)(1-p_5)$	J' → hr	$p_9(1-p_8)$	$1-p_{11}$
G → dV	p_{10}	p_6	g → dV'	p_{10}	p_{12}
V → bU	1		V' → bU'	1	
U → GJ	p_6p_7	p_4p_5	U' → GJ'	$(1-p_9)p_7$	0
U → HJ	$p_6(1-p_7)$	$p_4(1-p_5)$	U' → HJ'	$(1-p_9)(1-p_7)$	0
U → gJ	$(1-p_6)p_8$	$(1-p_4)p_5$	U' → gJ'	p_9p_8	p_{11}
U → hJ	$(1-p_6)(1-p_8)$	$(1-p_4)(1-p_5)$	U' → hJ'	$p_9(1-p_8)$	$1-p_{11}$
G → dW	$1-p_4-p_5-p_{10}$	$1-p_2-p_3-p_6$	g → dW'	$1-p_4-p_5-p_{10}$	$1-p_9-p_{10}-p_{12}$
W → bX	1		W' → bX'	1	
X → GU	p_6p_7	p_4p_5	X' → GU'	$(1-p_9)p_7$	0
X → HU	$p_6(1-p_7)$	$p_4(1-p_5)$	X' → HU'	$(1-p_9)(1-p_7)$	0
X → gU	$(1-p_6)p_8$	$(1-p_4)p_5$	X' → gU'	p_9p_8	p_{11}
X → hU	$(1-p_6)(1-p_8)$	$(1-p_4)(1-p_5)$	X' → hU'	$p_9(1-p_8)$	$1-p_{11}$
G → a	(1)		g → a	(1)	
H → e		p_2	h → d		p_9
e → B		1			

(continued on next page)

(continued from preceding page)

Rules	Latent mixing	Hidden	Rules	Latent mixing	Hidden
H → ei		p_3	h → di'		p_{10}
i → bj	1		i' → bj'	1	
j → Gr	$p_6 p_7$	$p_7 p_8$	j' → Gr	$(1 - p_9)p_7$	0
j → Hr	$p_6(1 - p_7)$	$p_7(1 - p_8)$	j' → Hr	$(1 - p_9)(1 - p_7)$	0
j → gr	$(1 - p_6)p_8$	$(1 - p_7)p_8$	j' → gr	$p_9 p_8$	p_{11}
j → hr	$(1 - p_6)(1 - p_8)$	$(1 - p_7)(1 - p_8)$	j' → hr	$p_9(1 - p_8)$	$1 - p_{11}$
H → ev		p_6	h → dv'		p_{12}
v → bu	1		v' → bu'	1	
u → Gj	$p_6 p_7$	p_6	u' → Gj'	$(1 - p_9)p_7$	0
u → Hj	$p_6(1 - p_7)$	$1 - p_6$	u' → Gj'	$(1 - p_9)(1 - p_7)$	0
u → gj	$(1 - p_6)p_8$	$(1 - p_7)p_8$	u' → gr	$p_9 p_8$	p_{11}
u → hj	$(1 - p_6)(1 - p_8)$	$(1 - p_7)(1 - p_8)$	u' → hr	$p_9(1 - p_8)$	$1 - p_{11}$
H → ew		$1 - p_2 - p_3 - p_6$	h → dw'		$1 - p_9 - p_{10} - p_{12}$
w → bx	1		w' → bx'	1	
x → Gu	$p_6 p_7$	p_6	x' → Gu'	$(1 - p_9)p_7$	0
x → Hu	$p_6(1 - p_7)$	$1 - p_6$	x' → Gu'	$(1 - p_9)(1 - p_7)$	0
x → gu	$(1 - p_6)p_8$	$(1 - p_7)p_8$	x' → gr	$p_9 p_8$	p_{11}
x → gu	$(1 - p_6)(1 - p_8)$	$(1 - p_7)(1 - p_8)$	x' → hr	$p_9(1 - p_8)$	$1 - p_{11}$
H → b	(1)		h → b	(1)	

Our choice of this grammar is motivated by results from binary visible state models without hidden states, is supported by model selection procedures (Akaike's information criterion), and obtains similar parameter estimates to the full binary visible state model with hidden states.
