# Supplementary material for: Parsing Social Network Survey Data from Hidden Populations Using Stochastic Context-Free Grammars
Source: PLoS One. 2009 Sep 7;4(9):e6777. doi: 10.1371/journal.pone.0006777 (PMC2734164; doi:10.1371/journal.pone.0006777)
Supplement: Text S2 — (0.04 MB PDF) [file pone.0006777.s004.pdf]

Supporting Text S2

Strings encoding RDS data.

Uniform recruitment strings (no assortment of respondents into visible groups):

1. `(.)`
2. `(.)`
3. `(.(.(.(..(.(.(.(:::)::).(:).(:))..).).(.(..(.(.(:::).).(:.(.:):))).(.(.(.(:))).(::).).(:.(:).):).(:)).(..(.(::).(:.(::).(::)).(:::)))).))`
4. `(.)`
5. `(.(...(.)))`
6. `(.(..(..(.(::).)).(.)))`
7. `(.(...(..(.(:::).(:::).))))`
8. `(.(.(...(.(.(:)))).(.(.(..(:).(.)).).).))`
9. `(.)`
10. `(.(.(.(..).(.(.(.(:)).(:)).(..(::))).(::)).(..:).))`
11. `(.)`
12. `(.)`
13. `(.(..(.(.(:)).(..).(:.(:::).(:::))).(.(..(.:.)).(.(:::).(:).(::)).)))`
14. `(.(.(...(:::)).(...).(:)))`
15. `(.(.(.(.(:::).(.(:::))).).(:).))`

Annotated by location (`A` = SVM, `B` = CM):

1. `(B)`
2. `(B)`
3. `(A(A(B(BB(A(A(B(bbb)ba)A(b)B(a))BA)B)B(B(BB(B(B(bbb)B)B(aA(Ab)b)))B(B(B(B(B(b)))B(bb)B)B(bB(b)A)b)B(b))B(BB(B(bb)B(bB(bb)A(ab))B(bbb))))B))`
4. `(B)`
5. `(B(BBB(B)))`
6. `(B(AB(BB(A(bb)A))B(B)))`
7. `(B(BBB(BB(B(bbb)B(bbb)B))))`
8. `(B(B(BBB(B(B(b)))B(A(B(BA(b)B(B))B)B)B))`

9. `(B)`
10. `(B(B(B(BB)B(A(B(B(b))B(b))B(BB(bb)))B(bb))B(BBb)B))`
11. `(B)`
12. `(A)`
13. `(A(AA(B(B(b))B(BA)B(aB(bbb)B(aba)))B(B(AB(AbB))B(B(bbb`
`)B(b)B(bb))B)))`
14. `(B(B(BBB(bba))B(BBB)B(b)))`
15. `(B(B(B(B(bbb)B(B(bbb)))B)B(b)B))`

Annotated by syphilis serostatus (`A` = seropositive, `B` = seronegative):

1. `(B)`
2. `(B)`
3. `(A(A(B(BB(A(A(B(bbb)ba)A(b)B(a))BA)B)B(B(BB(B(B(bbb)B)B`
`(aA(Ab)b)))B(B(B(B(B(b)))B(bb)B)B(bB(b)A)b)B(b))B(BB(B`
`(bb)B(bB(bb)A(ab))B(bbb))))B))`
4. `(B)`
5. `(B(BBB(B)))`
6. `(B(AB(BB(A(bb)A))B(B)))`
7. `(B(BBB(BB(B(bbb)B(bbb)B))))`
8. `(B(B(BBB(B(B(b)))B(A(B(BA(b)B(B))B)B)B))`
9. `(B)`
10. `(B(B(B(BB)B(A(B(B(b))B(b))B(BB(bb)))B(bb))B(BBb)B))`
11. `(B)`
12. `(A)`
13. `(A(AA(B(B(b))B(BA)B(aB(bbb)B(aba)))B(B(AB(AbB))B(B(bbb)`
`B(b)B(bb))B)))`
14. `(B(B(BBB(bba))B(BBB)B(b)))`
15. `(B(B(B(B(bbb)B(B(bbb)))B)B(b)B))`

Annotated by reporting drug-use at home in the past 6 months (`A` = yes, `B` = no):

1. `(B)`
2. `(B)`

3. (B(B(B(BB(B(B(B(aaa)ba)B(b)B(b))BA)A)B(A(BB(B(B(aab)A)A(bB(Ab)a)))B(B(B(B(A(a)))B(ab)B)B(bA(b)A)b)A(a))B(BB(A(aa)A(bB(ba)B(bb))B(bbb))))B))
4. (A)
5. (B(BBB(B)))
6. (B(BB(BB(B(aa)A))B(B)))
7. (B(BBB(BB(A(bba)B(abb)B))))
8. (B(B(BBA(A(B(a))))A(B(B(BB(b)B(B))A)B)B))
9. (A)
10. (B(A(B(AB)B(B(B(A(a))A(b))B(BA(aa)))A(aa))B(AAb)B))
11. (B)
12. (B)
13. (A(AA(B(B(a))B(BB)A(aA(aaa)B(bbb)))A(A(AB(AaB))B(B(bbb)B(b)B(ab))A)))
14. (A(B(ABB(aab))B(AAA)A(b)))
15. (B(B(B(B(bbb)B(B(bba)))B)B(b)B))

Annotated by 'drug-dealer' status (A = dealer, B = non-dealer):

1. (B)
2. (B)
3. (B(A(B(BB(B(B(B(bbb)bb)B(b)A(b))BA)B)B(B(BB(B(B(bab)B)B(bA(Bb)b)))B(B(B(B(B(b)))B(bb)B)B(aB(b)B)b)B(b))B(BB(A(ba)B(bB(bb)A(ba))B(bbb))))B))
4. (B)
5. (B(BBB(B)))
6. (A(BB(AB(A(ba)B))B(B)))
7. (B(BBB(BB(B(bbb)B(bbb)B))))
8. (B(B(BBB(B(B(b))))B(B(B(BB(b)A(B))B)B)B))
9. (B)
10. (B(B(B(BB)A(B(B(B(b))B(b))B(BB(bb)))B(bb))B(ABb)B))
11. (B)
12. (B)

13. `(B(BB(B(B(b))B(BB)B(bB(bbb)B(bbb)))B(B(BA(BbB))B(B(bbb) B(b)B(bb))B)))`

14. `(B(B(BBB(bbb))B(ABB)B(b)))`

15. `(B(B(A(B(bba)B(A(bba)))A)B(b)A))`

Annotated by reporting methamphetamine use (`A` = yes, `B` = no):

1. `(A)`

2. `(A)`

3. `(A(A(B(AA(A(A(A(aab)ba)A(a)A(a))AA)A)A(B(AA(B(A(aaa)A) A(bB(Aa)a)))A(A(A(B(A(a)))A(bb)A)A(aB(b)A)a)B(b))A(BA( A(aa)A(bB(aa)A(aa))A(bab))))A))`

4. `(A)`

5. `(A(AAA(A)))`

6. `(A(AA(BA(A(bb)A))A(B)))`

7. `(A(AAA(AA(B(aaa)A(aaa)A))))`

8. `(B(A(AAA(A(A(a)))A(A(A(BA(a)A(B))A)A)A))`

9. `(A)`

10. `(A(A(A(AA)A(B(A(A(a))A(a))A(AA(aa)))A(aa))A(BAa)A))`

11. `(A)`

12. `(A)`

13. `(A(AA(A(A(a))A(BB)A(aB(aaa)A(bab)))A(A(AA(AaB)) A(B(aaa)A(a)A(bb))A)))`

14. `(A(A(ABA(baa))A(BAA)A(a)))`

15. `(B(A(B(A(aaa)A(A(aab)))A)B(a)A))`
