# Supplementary material for: Parsing Social Network Survey Data from Hidden Populations Using Stochastic Context-Free Grammars
Source: PLoS One. 2009 Sep 7;4(9):e6777. doi: 10.1371/journal.pone.0006777 (PMC2734164; doi:10.1371/journal.pone.0006777)
Supplement: Table S1 — Model parameter estimates are grouped by ‘visible’ attributes of respondents (location, syphilis serostatus, drug-dealing, drug-use at home, and use of methamphetamine). Each model of recruitment was evaluated using a custom SCFG, comprising the rules listed with each hypothesis: 2-visible, full recruitment model with binary visible states; constrain mix, constrained mixing rates between visible states to be equal; constrain N (M2 baseline model), constrained distribution in the number of recruits per respondent to be equal; constrain both (M2 C), constrained both mixing rates and distributions in the number of recruits; (5) dependent mixing (M2 D), permitted mixing rates to be dependent on the number of recruits; (6) latent-mixing (M2×2 L), mixing on latent variables, independent of the visible state of the recruiter; and (7) add hidden (M2×2 H), addition of hidden states representing variation in mixing rates and number of recruits over time. Production rules for each grammar are presented in non-reduced form to conserve space. Column header abbreviations are defined as follows: MLE = maximum likelihood estimate of model parameter; lower, upper 95% = lower and upper limits of the 95% confidence interval, estimated using likelihood profiling; log L, log-transformed likelihood of the model; df = degrees of freedom, i.e., number of free parameters in the model; AIC = Akaike's Information Criterion. (0.09 MB PDF) [file pone.0006777.s005.pdf]

LOCATION	MLE	Lower 95%	Upper 95%	log L	df	AIC
**2-visible**				-239.281	9	496.562
S->(G)	0.800	0.560	0.946			
S->(H)	0.200	0.054	0.440			
G->A	0.351	0.267	0.441			
G->A(X)	0.149	0.092	0.222			
G->A(XX)	0.175	0.113	0.252			
G->A(XXX)	0.325	0.243	0.414			
X->G	0.958	0.921	0.982			
X->H	0.042	0.018	0.079			
H->B	0.441	0.283	0.472			
H->B(Y)	0.176	0.074	0.326			
H->B(YY)	0.206	0.094	0.360			
H->B(YYY)	0.176	0.074	0.326			
Y->G	0.053	0.009	0.154			
Y->H	0.947	0.846	0.991			
**constrain mix**				-307.877	8	631.754
S->(G)	0.800	0.560	0.946			
S->(H)	0.200	0.054	0.440			
G->A	0.351	0.267	0.441			
G->A(X)	0.149	0.092	0.222			
G->A(XX)	0.175	0.113	0.252			
G->A(XXX)	0.325	0.243	0.414			
X->G	0.791	0.732	0.843			
X->H	0.209	0.157	0.268			
H->B	0.441	0.283	0.608			
H->B(Y)	0.176	0.074	0.326			
H->B(YY)	0.206	0.094	0.360			
H->B(YYY)	0.176	0.074	0.326			
**constrain N**				-240.788	6	493.576
S->(G)	0.800	0.560	0.946			
S->(H)	0.200	0.054	0.440			
G->A	0.372	0.296	0.451			
G->A(X)	0.155	0.103	0.219			
G->A(XX)	0.182	0.126	0.250			
G->A(XXX)	0.291	0.221	0.367			
X->G	0.958	0.921	0.982			
X->H	0.042	0.018	0.079			
Y->G	0.053	0.009	0.154			
Y->H	0.947	0.846	0.991			
**constrain both**				-309.384	5	628.768
S->(G)	0.800	0.560	0.946			
S->(H)	0.200	0.054	0.440			
G->A	0.372	0.296	0.451			
G->A(X)	0.155	0.103	0.219			
G->A(XX)	0.182	0.126	0.250			
G->A(XXX)	0.291	0.221	0.367			
X->G	0.791	0.732	0.843			
X->H	0.209	0.157	0.268			
**dependent mixing**				-238.662	7	491.324
S->(G)	0.800	0.560	0.946			
S->(H)	0.200	0.054	0.440			
G->A	0.372	0.296	0.451			
G->A(X)	0.155	0.103	0.219			
G->A(XX)	0.182	0.126	0.250			
G->A(XXX)	0.291	0.221	0.367			
X->G	0.996	0.991	0.998			
X->H	0.004	0.002	0.009			
XX->GX	0.984	0.984	0.984			
XX->HX	0.016	0.011	0.023			
XXX->GXX	0.943	0.940	0.945			
XXX->HXX	0.057	0.026	0.103			
Y->G	0.167	0.031	0.411			
Y->H	0.833	0.589	0.969			
YY->GY	0.052	0.038	0.075			
YY->HY	0.948	0.947	0.949			
YYY->GYY	0.015	0.010	0.022			
YYY->HYY	0.985	0.985	0.985			
Gfactor						
**latent mixing**				-309.144	10	638.288
S->(G)	0.000	0.000	0.713			
S->(H)	0.200	0.054	0.440			
S->(g)	0.800	0.560	0.946			
S->(h)	0.000	0.000	0.272			
G->A	0.372	0.296	0.451			
G->A(X)	0.155	0.103	0.219			
G->A(XX)	0.182	0.126	0.250			
G->A(XXX)	0.291	0.221	0.367			
X->G	0.000	0.000	0.473			
X->H	0.000	0.000	0.324			
X->g	0.775	0.241	0.787			
X->h	0.225	0.022	0.271			
x->G	0.081	0.000	0.229			
x->H	0.000	0.000	0.127			
x->g	0.712	0.642	0.742			
x->h	0.207	0.135	0.267			
**add hidden**				-234.651	13	495.302
S->(g)	0.800	0.560	0.946			
S->(h)	0.200	0.054	0.440			
G->A	0.368	0.288	0.453			
G->A(X)	0.176	0.117	0.247			
G->A(XX)	0.197	0.135	0.271			
G->A(XXX)	0.259	0.189	0.339			
X->G	0.966	0.965	0.967			
X->H	0.034	0.266	0.678			
Y->G	0.078	0.014	0.196			
Y->H	0.922	0.909	0.927			
g->A	0.401	0.187	0.644			
g->A(x)	0.000	0.000	0.111			
g->A(xx)	0.067	0.004	0.263			
g->A(xxx)	0.531	0.295	0.760			
x->G	0.900	0.863	0.878			
x->H	0.100	0.012	0.236			
x->g	0.000	0.000	0.261			
x->h	0.000	0.000	0.006			
y->G	0.000	0.000	0.063			
y->H	0.791	0.556	0.827			
y->g	0.000	0.000	0.120			
y->h	0.209	0.017	0.386			

SYPHILIS	MLE	Lower 95%	Upper 95%	log L	df	AIC
**2-visible**				-282.068	9	582.136
S->(G)	0.200	0.054	0.440			
S->(H)	0.800	0.560	0.946			
G->A	0.435	0.247	0.636			
G->A(X)	0.087	0.015	0.245			
G->A(XX)	0.261	0.113	0.459			
G->A(XXX)	0.217	0.084	0.410			
X->G	0.276	0.137	0.453			
X->H	0.724	0.547	0.863			
H->B	0.360	0.279	0.446			
H->B(Y)	0.168	0.110	0.240			
H->B(YY)	0.168	0.110	0.240			
H->B(YYY)	0.304	0.228	0.388			
Y->G	0.113	0.072	0.165			
Y->H	0.887	0.835	0.928			
**constrain mix**				-284.437	8.000	584.874
S->(G)	0.200	0.054	0.440			
S->(H)	0.800	0.560	0.946			
G->A	0.435	0.247	0.636			
G->A(X)	0.087	0.015	0.245			
G->A(XX)	0.261	0.113	0.459			
G->A(XXX)	0.217	0.084	0.410			
X->G	0.136	0.094	0.187			
X->H	0.864	0.813	0.906			
H->B	0.360	0.279	0.446			
H->B(Y)	0.168	0.110	0.240			
H->B(YY)	0.168	0.110	0.240			
H->B(YYY)	0.304	0.228	0.388			
Y->G						
Y->H						
**constrain N**				-283.369	6.000	578.738
S->(G)	0.200	0.054	0.440			
S->(H)	0.800	0.560	0.946			
G->A	0.372	0.296	0.451			
G->A(X)	0.155	0.103	0.219			
G->A(XX)	0.182	0.126	0.250			
G->A(XXX)	0.291	0.221	0.367			
X->G	0.276	0.137	0.453			
X->H	0.724	0.547	0.863			
H->B						
H->B(Y)						
H->B(YY)						
H->B(YYY)						
Y->G	0.113	0.072	0.165			
Y->H	0.887	0.835	0.928			
**constrain both**				-285.738	5	581.476
S->(G)	0.200	0.054	0.440			
S->(H)	0.800	0.560	0.946			
G->A	0.372	0.296	0.451			
G->A(X)	0.155	0.103	0.219			
G->A(XX)	0.182	0.126	0.250			
G->A(XXX)	0.291	0.221	0.367			
X->G	0.136	0.094	0.187			
X->H	0.864	0.813	0.906			
**latent mixing**				-281.019	10	582.038
S->(G)	0.200	0.054	0.440			
S->(H)	0.000	0.000	0.439			
S->(g)	0.000	0.000	0.188			
S->(h)	0.800	0.560	0.946			
G->A	0.372	0.296	0.451			
G->A(X)	0.155	0.103	0.219			
G->A(XX)	0.182	0.126	0.250			
G->A(XXX)	0.291	0.221	0.367			
X->G	0.279	0.152	0.411			
X->H	0.476	0.319	0.571			
X->g	0.003	0.000	0.033			
X->h	0.243	0.098	0.373			
x->G	0.048	0.012	0.128			
x->H	0.081	0.024	0.174			
x->g	0.009	0.000	0.068			
x->h	0.862	0.834	0.873			
**add hidden**				-275.94	13	577.88
S->(g)	0.200	0.054	0.440			
S->(h)	0.800	0.560	0.946			
G->A	0.375	0.292	0.464			
G->A(X)	0.186	0.124	0.261			
G->A(XX)	0.204	0.139	0.281			
G->A(XXX)	0.235	0.163	0.318			
X->G	0.245	0.111	0.380			
X->H	0.755	0.687	0.787			
Y->G	0.133	0.087	0.185			
Y->H	0.867	0.858	0.873			
g->A	0.353	0.167	0.579			
g->A(x)	0.000	0.000	0.097			
g->A(xx)	0.072	0.005	0.242			
g->A(xxx)	0.574	0.354	0.776			
x->G	0.121	0.021	0.098			
x->H	0.224	0.009	0.538			
x->g	0.229	0.000	0.484			
x->h	0.426	0.000	0.304			
y->G	0.032	0.002	0.096			
y->H	0.873	0.819	0.887			
y->g	0.003	0.000	0.047			
y->h	0.092	0.181	0.390			

DEALER	MLE	Lower 95%	Upper 95%	log L	df	AIC
**2-visible**				-272.552	9	563.104
S->(G)	0.067	0.004	0.262			
S->(H)	0.933	0.738	0.996			
G->A	0.333	0.148	0.563			
G->A(X)	0.111	0.019	0.305			
G->A(XX)	0.333	0.148	0.563			
G->A(XXX)	0.222	0.075	0.443			
X->G	0.154	0.051	0.322			
X->H	0.846	0.678	0.949			
H->B	0.377	0.297	0.462			
H->B(Y)	0.162	0.105	0.231			
H->B(YY)	0.162	0.105	0.231			
H->B(YYY)	0.300	0.226	0.382			
Y->G	0.111	0.071	0.162			
Y->H	0.889	0.838	0.929			
**constrain mix**				-272.739	8	561.478
S->(G)	0.067	0.004	0.262			
S->(H)	0.933	0.738	0.996			
G->A	0.333	0.148	0.563			
G->A(X)	0.111	0.019	0.305			
G->A(XX)	0.333	0.148	0.563			
G->A(XXX)	0.222	0.075	0.443			
X->G	0.117	0.078	0.165			
X->H	0.883	0.835	0.922			
H->B	0.377	0.297	0.462			
H->B(Y)	0.162	0.105	0.231			
H->B(YY)	0.162	0.105	0.231			
H->B(YYY)	0.300	0.226	0.382			
Y->G						
Y->H						
**constrain N**				-273.975	6	559.95
S->(G)	0.067	0.004	0.262			
S->(H)	0.933	0.738	0.995			
G->A	0.372	0.296	0.451			
G->A(X)	0.155	0.103	0.219			
G->A(XX)	0.182	0.126	0.250			
G->A(XXX)	0.291	0.221	0.367			
X->G	0.154	0.051	0.322			
X->H	0.846	0.678	0.949			
Y->G	0.111	0.071	0.162			
Y->H	0.889	0.838	0.929			
**constrain both**				-274.162	5	558.324
S->(G)	0.067	0.004	0.262			
S->(H)	0.933	0.738	0.995			
G->A	0.372	0.296	0.451			
G->A(X)	0.155	0.103	0.219			
G->A(XX)	0.182	0.126	0.250			
G->A(XXX)	0.291	0.221	0.367			
X->G	0.117	0.078	0.165			
X->H	0.883	0.835	0.922			
**latent mixing**				-273.688	10	567.376
S->(G)	0.000	0.000	0.190			
S->(H)	0.000	0.000	0.856			
S->(g)	0.067	0.004	0.262			
S->(h)	0.933	0.738	0.991			
G->A	0.372	0.296	0.451			
G->A(X)	0.155	0.103	0.219			
G->A(XX)	0.182	0.126	0.250			
G->A(XXX)	0.291	0.221	0.367			
X->G	0.023	0.000	0.140			
X->H	0.237	0.000	0.557			
X->g	0.116	0.000	0.225			
X->h	0.625	0.000	0.711			
x->G	0.089	0.000	0.148			
x->H	0.911	0.000	0.915			
x->g	0.000	0.000	0.204			
x->h	0.000	0.000	0.480			
**add hidden**				-268.141	13	562.282
S->(g)	0.067	0.004	0.262			
S->(h)	0.933	0.738	0.995			
G->A	0.365	0.283	0.452			
G->A(X)	0.181	0.121	0.255			
G->A(XX)	0.202	0.138	0.278			
G->A(XXX)	0.252	0.179	0.334			
p3						
p4						
p5						
p6						
X->G	0.186	0.070	0.322			
X->H	0.814	0.767	0.834			
Y->G	0.115	0.073	0.164			
Y->H	0.885	0.878	0.890			
g->A	0.412	0.198	0.648			
g->A(x)	0.000	0.000	0.108			
g->A(xx)	0.063	0.004	0.244			
g->A(xxx)	0.525	0.296	0.749			
x->G	0.000	0.000	0.080			
x->H	1.000	1.000	1.000			
x->g	0.000	0.000	0.227			
x->h	0.000	0.000	0.324			
y->G	0.076	0.019	0.123			
y->H	0.732	0.593	0.786			
y->g	0.018	0.000	0.108			
y->h	0.174	0.230	0.386			

HOME-USE	MLE	Lower 95%	Upper 95%	log L	df	AIC
**2-visible**				-333.597	9	685.194
S->(G)	0.267	0.092	0.515			
S->(H)	0.733	0.485	0.908			
G->A	0.455	0.313	0.601			
G->A(X)	0.182	0.088	0.312			
G->A(XX)	0.136	0.057	0.257			
G->A(XXX)	0.227	0.121	0.364			
X->G	0.520	0.383	0.655			
X->H	0.480	0.345	0.617			
H->B	0.337	0.250	0.431			
H->B(Y)	0.144	0.086	0.220			
H->B(YY)	0.202	0.133	0.286			
H->B(YYY)	0.317	0.233	0.410			
Y->G	0.301	0.233	0.376			
Y->H	0.699	0.624	0.767			
**constrain mix**				-337.438	8	690.876
S->(G)	0.267	0.092	0.515			
S->(H)	0.733	0.485	0.908			
G->A	0.455	0.313	0.601			
G->A(X)	0.182	0.088	0.312			
G->A(XX)	0.136	0.057	0.257			
G->A(XXX)	0.227	0.121	0.364			
X->G	0.354	0.291	0.421			
X->H	0.646	0.579	0.709			
H->B	0.337	0.250	0.431			
H->B(Y)	0.144	0.086	0.220			
H->B(YY)	0.202	0.133	0.286			
H->B(YYY)	0.317	0.233	0.410			
**constrain N**				-335.128	6	682.256
S->(G)	0.267	0.092	0.515			
S->(H)	0.733	0.485	0.908			
G->A	0.372	0.296	0.451			
G->A(X)	0.155	0.103	0.219			
G->A(XX)	0.182	0.126	0.250			
G->A(XXX)	0.291	0.221	0.367			
X->G	0.520	0.383	0.655			
X->H	0.480	0.345	0.617			
Y->G	0.301	0.233	0.376			
Y->H	0.699	0.624	0.767			
**constrain both**				-338.97	5	687.94
S->(G)	0.267	0.092	0.515			
S->(H)	0.733	0.485	0.908			
G->A	0.372	0.296	0.451			
G->A(X)	0.155	0.103	0.219			
G->A(XX)	0.182	0.126	0.250			
G->A(XXX)	0.291	0.221	0.367			
X->G	0.354	0.291	0.421			
X->H	0.646	0.579	0.709			
**latent mixing**				-331.919	10	683.838
S->(G)	0.267	0.092	0.515			
S->(H)	0.000	0.000	0.284			
S->(g)	0.000	0.000	0.207			
S->(h)	0.733	0.485	0.908			
G->A	0.372	0.296	0.451			
G->A(X)	0.155	0.193	0.327			
G->A(XX)	0.182	0.126	0.250			
G->A(XXX)	0.291	0.221	0.367			
X->G	0.532	0.421	0.610			
X->H	0.375	0.242	0.465			
X->g	0.007	0.000	0.046			
X->h	0.086	0.005	0.213			
x->G	0.144	0.068	0.246			
x->H	0.102	0.039	0.188			
x->g	0.055	0.000	0.149			
x->h	0.699	0.625	0.741			
**add hidden**				-327.539	13	681.078
S->(g)	0.267	0.092	0.515			
S->(h)	0.733	0.485	0.908			
G->A	0.368	0.290	0.452			
G->A(X)	0.173	0.115	0.243			
G->A(XX)	0.196	0.134	0.268			
G->A(XXX)	0.263	0.193	0.342			
p3						
p4						
p5						
p6						
X->G	0.500	0.415	0.563			
X->H	0.500	0.416	0.563			
Y->G	0.331	0.276	0.381			
Y->H	0.669	0.640	0.692			
g->A	0.400	0.183	0.649			
g->A(x)	0.000	0.000	0.120			
g->A(xx)	0.067	0.000	0.262			
g->A(xxx)	0.533	0.291	0.765			
x->G	0.667	0.445	0.457			
x->H	0.333	0.027	0.519			
x->g	0.000	0.000	0.400			
x->h	0.000	0.000	0.044			
y->G	0.100	0.019	0.163			
y->H	0.900	0.819	0.908			
y->g	0.000	0.000	0.094			
y->h	0.000	0.401	0.420			

METH	MLE	Lower 95%	Upper 95%	log L	df	AIC
**2-visible**				-305.188	9	628.376
S->(G)	0.867	0.642	0.977			
S->(H)	0.133	0.023	0.358			
G->A	0.364	0.281	0.452			
G->A(X)	0.157	0.100	0.229			
G->A(XX)	0.174	0.113	0.248			
G->A(XXX)	0.306	0.264	0.391			
X->G	0.767	0.701	0.826			
X->H	0.233	0.174	0.299			
H->B	0.407	0.237	0.595			
H->B(Y)	0.148	0.049	0.311			
H->B(YY)	0.222	0.095	0.400			
H->B(YYY)	0.222	0.095	0.400			
Y->G	0.912	0.787	0.977			
Y->H	0.088	0.023	0.213			
**constrain mix**				-307.287	8	630.574
S->(G)	0.867	0.642	0.977			
S->(H)	0.133	0.023	0.358			
G->A	0.364	0.281	0.452			
G->A(X)	0.157	0.100	0.229			
G->A(XX)	0.174	0.113	0.248			
G->A(XXX)	0.306	0.228	0.391			
X->G	0.791	0.732	0.843			
X->H	0.209	0.157	0.268			
H->B	0.407	0.237	0.595			
H->B(Y)	0.148	0.049	0.311			
H->B(YY)	0.222	0.095	0.400			
H->B(YYY)	0.222	0.095	0.062			
**constrain N**				-305.669	6	623.338
S->(G)	0.867	0.642	0.977			
S->(H)	0.133	0.023	0.358			
G->A	0.372	0.296	0.451			
G->A(X)	0.155	0.103	0.219			
G->A(XX)	0.182	0.126	0.250			
G->A(XXX)	0.291	0.221	0.367			
X->G	0.767	0.701	0.826			
X->H	0.233	0.174	0.299			
Y->G	0.912	0.787	0.977			
Y->H	0.088	0.189	0.213			
**constrain both**				-307.768	5	625.536
S->(G)	0.867	0.642	0.977			
S->(H)	0.133	0.023	0.358			
G->A	0.372	0.296	0.451			
G->A(X)	0.155	0.103	0.219			
G->A(XX)	0.182	0.126	0.250			
G->A(XXX)	0.291	0.221	0.367			
X->G	0.791	0.732	0.843			
X->H	0.209	0.157	0.268			
**latent mixing**				-300.509	10	621.018
S->(G)	0.000	0.000	0.370			
S->(H)	0.107	0.005	0.337			
S->(g)	0.867	0.642	0.977			
S->(h)	0.026	0.000	0.248			
G->A	0.372	0.296	0.451			
G->A(X)	0.155	0.193	0.327			
G->A(XX)	0.182	0.126	0.250			
G->A(XXX)	0.291	0.221	0.367			
X->G	0.000	0.000	0.158			
X->H	0.762	0.691	0.777			
X->g	0.231	0.000	0.289			
X->h	0.007	0.000	0.239			
x->G	0.000	0.000	0.184			
x->H	1.000	1.000	1.000			
x->g	0.000	0.000	0.073			
x->h	0.000	0.000	0.073			
**add hidden**				-294.872	13	615.744
S->(g)	0.867	0.642	0.977			
S->(h)	0.133	0.023	0.358			
G->A	0.368	0.288	0.453			
G->A(X)	0.175	0.117	0.247			
G->A(XX)	0.197	0.135	0.271			
G->A(XXX)	0.259	0.189	0.339			
p3						
p4						
p5						
p6						
X->G	0.731	0.709	0.748			
X->H	0.269	0.216	0.321			
Y->G	0.929	0.918	0.933			
Y->H	0.071	0.013	0.181			
g->A	0.400	0.184	0.645			
g->A(x)	0.000	0.000	0.118			
g->A(xx)	0.068	0.004	0.262			
g->A(xxx)	0.533	0.294	0.763			
x->G	0.955	0.934	0.957			
x->H	0.000	0.000	0.082			
x->g	0.045	0.000	0.238			
x->h	0.000	0.000	0.000			
y->G	0.713	0.432	0.608			
y->H	0.143	0.003	0.392			
y->g	0.121	0.000	0.467			
y->h	0.024	0.134	0.355			
